# Insights Into Macrophage Ferroptosis: Implications for Atherosclerosis

**DOI:** 10.1111/cpr.70154

**Published:** 2025-12-29

**Authors:** Xiehui Chen, Xiangbo Liu, Changchun Zeng

**Affiliations:** ^1^ Department of Geriatrics Shenzhen Longhua District Central Hospital Shenzhen China; ^2^ Key Laboratory of Personalized Precision Treatment for Elderly Coronary Heart Disease Shenzhen China; ^3^ Department of Cardiovascular Medicine Shenzhen Longhua District Central Hospital Shenzhen China; ^4^ Department of Medical Laboratory Shenzhen Longhua District Central Hospital Shenzhen China

**Keywords:** atherosclerosis, ferroptosis, iron metabolism, macrophage, treatment

## Abstract

Atherosclerosis remains a significant global health challenge, arising from the complex interactions among dysregulated lipid metabolism, chronic inflammation and immune activation. Ferroptosis, marked by lipid peroxide buildup dependent on iron, is gaining recognition as a modulator of macrophage activity in atherosclerosis. Macrophages are the pivotal orchestrators of chronic inflammation and atherosclerotic plaque formation. The marked heterogeneity and plasticity of macrophages within plaques dynamically shape the local microenvironment, contributing to phenomena such as lipid overload, cytokine overactivation, hypoxia, and programmed cell death. This review examines how dysregulated iron handling, lipid metabolism, and redox imbalances synergise to induce macrophage ferroptosis in atherosclerosis. Moreover, ferroptosis contributes to the development and progression of atherosclerosis by causing dysfunction in vascular smooth muscle cells (VSMCs), vascular endothelial cells (VECs), and macrophages, thereby promoting plaque formation and instability. Furthermore, macrophages are intricately linked to ferroptosis, with this iron‐dependent cell death enhancing oxidative stress and inflammatory pathways. Macrophage ferroptosis drives plaque progression and destabilisation, ultimately heightening the risk of rupture and cardiovascular events. By inhibiting macrophage ferroptosis, it may be possible to reduce oxidative stress and inflammation, stabilise atherosclerotic plaques, and ultimately lower the risk of cardiovascular events. This review highlights the therapeutic potential of targeting macrophage ferroptosis for the treatment of atherosclerosis.

## Graphical abstract

Macrophage ferroptosis drives atherosclerosis by enhancing oxidative stress and inflammation, accelerating plaque progression and instability. Targeting macrophage ferroptosis presents a promising therapeutic strategy for atherosclerosis treatment.

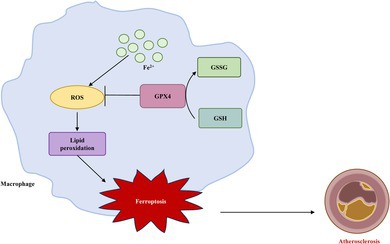

**Abbreviations**
4‐HNE: 4‐hydroxynonenal
AA: arachidonic acid
AdA: adrenic acid
FALs: fatty alcohols
FAR1: fatty acyl‐CoA reductase 1
FPN: ferroportin 1
GSH: glutathione
LDL: low‐density lipoprotein
LDs: lipid droplets
LOXs: lipoxygenases
LPO: lipid peroxidation
MDA: malondialdehyde
MMPs: matrix metalloproteinases
MUFAs: monounsaturated fatty acids
NOX: NADPH oxidase
NTBI: non‐transferrin‐bound iron
ox‐LDL: oxidised low‐density lipoprotein
PLOOH: phospholipid hydroperoxides
PUFA: polyunsaturated fatty acids
RBCs: red blood cells
ROS: reactive oxygen species
SCD1: stearoyl‐CoA desaturase 1
SFAs: saturated fatty acids
SOD: superoxide dismutase
TFR1: transferrin receptor 1
VECs: vascular endothelial cells
VSMCs: vascular smooth muscle cells

## 1 Introduction

Atherosclerosis, the primary condition affecting the cardiovascular system, is marked by lipid accumulation, plaque formation, and arterial inflammation, which may result in clinical manifestations like stroke and myocardial infarction [1]. The disease pathogenesis is a multi‐faceted process involving not only fibrosis and lipid deposition, but also a complex interaction of VSMC activation, VEC impairment, and immune cell recruitment, culminating in the genesis and propagation of lesions [2]. Despite the recognition of risk factors like hypertension, smoking and hyperlipidemia, research points to the crucial involvement of diverse cell death pathways in the pathogenesis and evolution of atherosclerosis [3]. Notably, ferroptosis has emerged as a potential key contributor to the pathophysiological mechanisms underlying atherosclerosis [4, 5].

Iron accumulation within atherosclerotic plaques, indicative of local disruption of iron homeostasis, is associated with plaque instability and disease progression [6]. Augmented iron levels, commonly linked to lipid dysregulation and inflammation, foster the production of lipid peroxidation (LPO) and reactive oxygen species (ROS). Additionally, lipid metabolic dysregulation in the arterial wall, especially the accumulation of oxidised lipids, triggers ferroptosis [7]. Disruption of redox homeostasis further exacerbates oxidative stress, thereby advancing the ferroptotic pathway [8]. Distinct from other cell death modalities, ferroptosis is characterised by an iron‐dependent accumulation of lipid peroxides that ultimately leads to membrane damage and cell death [9]. This process is intricately connected to the management of iron and lipids, as well as redox disequilibrium, making it notably pertinent in the oxidative, inflammatory setting of atherosclerotic plaques. The evolution of atherosclerosis is driven by diverse cell types, notably VSMCs, VECs and macrophages, each exerting a specific influence on plaque development and its propensity for rupture [10]. As central players in the innate immune response, macrophages significantly contribute to the development of atherosclerotic plaques [11]. These cells migrate into the arterial wall, ingest oxidised lipids, and drive both the inflammatory reaction and plaque development. Several factors, including oxidative stress, altered lipid metabolism, and excessive iron accumulation, can modulate the ferroptotic process in macrophages within atherosclerotic lesions [12]. This review presents an updated perspective on ferroptosis in atherosclerosis, with particular emphasis on its effects on macrophage biology and dysfunction. Deciphering the complexities of ferroptosis within macrophages is essential for progressing novel therapeutic strategies.

## 2 Iron Homeostasis in Atherosclerosis

Iron is an indispensable micronutrient involved in various biological functions, including cytochrome enzyme activity, DNA replication, myoglobin and haemoglobin synthesis, and mitochondrial processes [13]. Iron homeostasis is strictly controlled to maintain adequate iron levels for crucial biological processes, while simultaneously mitigating the toxic effects of excessive iron accumulation [14]. Cellular iron uptake primarily occurs through the binding of transferrin to transferrin receptor 1 (TFR1), which increases intracellular iron levels and upregulates ferritin synthesis. Elevated TFR1 expression is associated with increased susceptibility to ferroptosis due to enhanced iron accumulation and LPO [15]. STEAP3, an endosomal ferrireductase, catalyses the reduction of Fe^3+^ to Fe^2+^, enabling subsequent recognition and transport of Fe^2+^ into the cell by DMT1 [6]. Fe^2+^ can be exported via ferroportin, stored in ferritin, incorporated into enzyme and heme synthesis, or participate in other cellular activities, including oxidative metabolism. The activity of ferroportin is inhibited by hepcidin, which binds to ferroportin and induces its internalisation and lysosomal degradation. This prevents iron export from enterocytes and macrophages, thereby lowering serum iron levels while elevating intracellular iron stores [16]. A reduction in hepcidin levels due to iron deficiency promotes iron absorption and stimulates the release of stored iron [17]. The excessive rise in ferritin levels is associated with accelerated early atherosclerosis, reflecting disrupted iron balance that triggers oxidative stress and inflammation, key factors in atherosclerotic plaque progression. Hepcidin drives erastin‐triggered ferroptosis via intracellular iron accumulation, a process mediated by ferroportin's conformational shift and its degradation [18]. Intracellular labile ferrous iron can participate in the Fenton reaction with hydrogen peroxide, thereby generating hydroxyl radicals [9]. The buildup of ROS can oxidise polyunsaturated fatty acids (PUFA) bound to phospholipids, forming lipid hydroperoxides (PL‐PUFA‐OOH). This LPO impairs membrane stability and triggers ferroptosis. System Xc‐, consisting of SLC7A11 and SLC3A2, mediates the efflux of glutamate and the influx of cystine to preserve redox equilibrium, and its malfunction could impair the glutathione (GSH) equilibrium [19]. Cystine contributes to the production of GSH, an antioxidant crucial for combating oxidative stress. GPX4 catalyses the GSH‐dependent reduction of phospholipid hydroperoxides, such as PUFA‐PE‐OOH, to PUFA‐PE‐OH, producing GSSG and H_2_O as byproducts. This reaction protects cellular membranes from LPO‐induced damage and acts as a central cellular defence against ferroptosis. PUFAs like adrenic acid (AdA) and arachidonic acid (AA) undergo conversion into CoA derivatives (PUFA‐CoAs), followed by enzymatic modification via LPCAT3, enabling their incorporation into cellular membrane phospholipids [20]. Upon oxidation of PUFAs in membrane phospholipids, lipoxygenases (LOXs) catalyse their conversion into hydroperoxides (PL‐PUFA(PE)‐OOH), triggering the ferroptotic signalling pathway [21]. Iron overload can enhance non‐transferrin‐bound iron (NTBI) uptake via SLC39A14 in macrophages and hepatocytes, thereby exacerbating intracellular iron accumulation, oxidative stress, and cytotoxicity. The rise in NTBI is linked to vascular dysfunction, a fundamental process in atherosclerotic development [9, 22].

## 3 Ferroptosis: A Novel Player in Atherosclerosis

**FIGURE 1:**
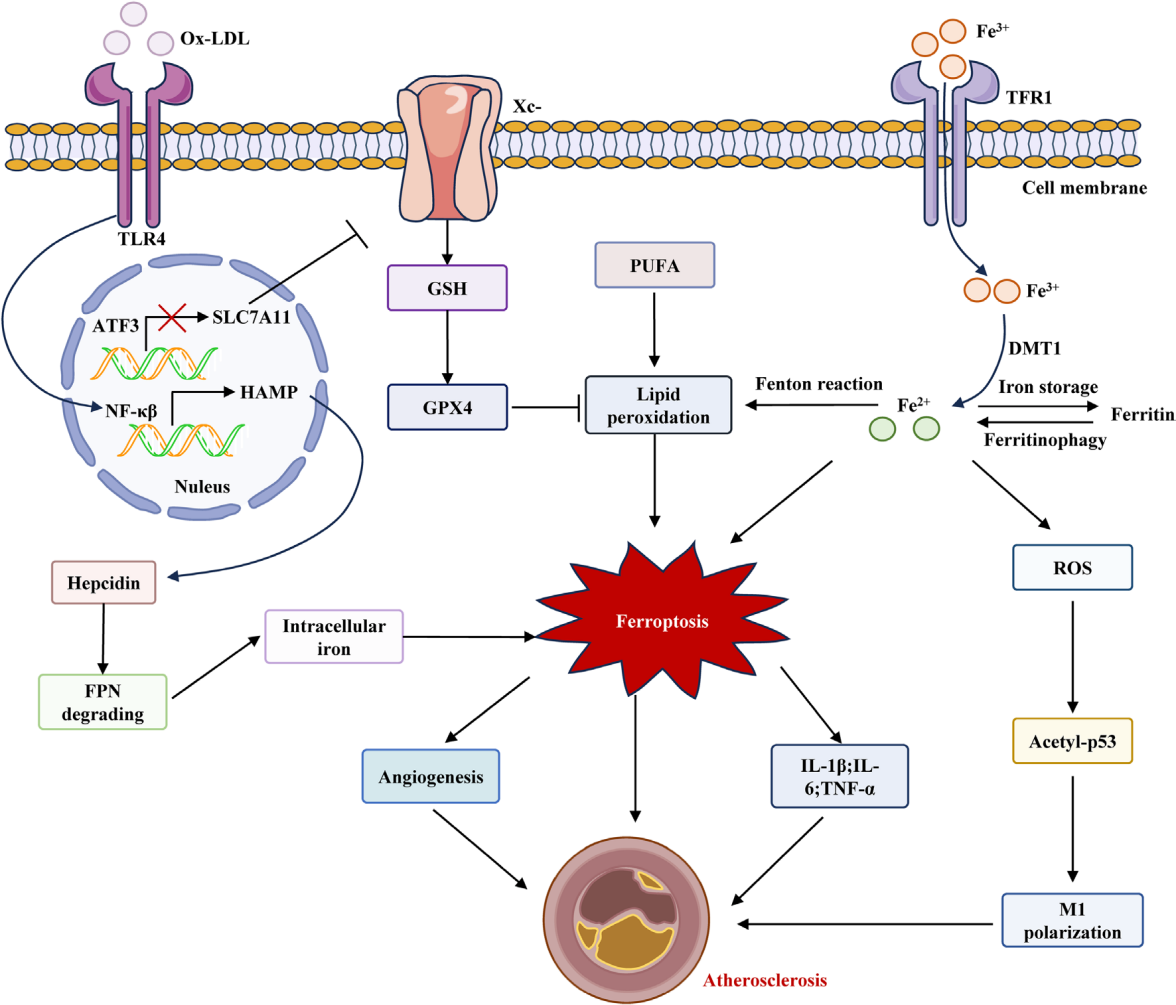
Molecular pathways linking ferroptosis to atherosclerosis. Iron accumulation and ox‐LDL, pivotal in lipid peroxidation, trigger ferroptosis and propel atherosclerosis. Fe^3+^ is transported into cells via TFR1 and transformed into Fe^2+^ via the Fenton reaction. An excess of Fe^2+^ results in cellular iron overload, promoting ferroptosis and boosting ROS generation within cells, which triggers M1 macrophage polarisation via the acetyl‐p53 pathway, thus contributing to AS development. Ox‐LDL boosts hepcidin expression via the TLR4/NF‐κB pathway, leading to FPN degradation, thus increasing intracellular iron and ferroptosis. Ox‐LDL also synergizes with iron in lipid peroxidation, with Gpx4 as the principal defence. The relationship between ferroptosis and atherosclerosis might be modulated through angiogenesis and inflammation pathways.

Ferroptosis, a form of regulated cell death driven by iron‐dependent LPO, has emerged as an important contributing mechanism in the progression and destabilisation of atherosclerotic plaques [23]. The mechanism is typified by impaired oxidative‐antioxidant equilibrium (Figure 1). Ferroptosis involves pronounced iron‐driven LPO, heightened oxidative stress and compromised antioxidant defences, exerting significant effects on vascular stability, plaque evolution and atherosclerosis advancement [4, 19].

### 3.1 Iron Metabolism and Ferroptosis

The regulation of iron levels in cells involves uptake through the plasma membrane, compartmental redistribution, and exportation [24]. Dietary iron is assimilated by intestinal epithelial cells and delivered to the bloodstream. Moreover, macrophages engulf old red blood cells (RBCs), resulting in increased iron concentrations in the bloodstream. The TFR1‐mediated internalisation of ferric iron constitutes a pivotal process in cellular iron metabolism. Within endosomes, the ferric ion is then reduced to ferrous iron by ferric reductases, such as STEAP3, before its translocation into the cytosol via DMT1 [25]. The remaining ferrous iron either enters the labile iron pool or is stored in ferritin. Impaired ferroportin 1 (FPN)–mediated iron export leads to intracellular iron overload, which heightens susceptibility to ferroptosis. The harmful effects of labile iron ions are primarily mediated by the Fenton reaction, which generates highly reactive hydroxyl radicals, leading to oxidative damage to DNA, lipids, and proteins. NCOA4 mediates ferritinophagy by delivering ferritin to autophagosomes for degradation, triggering the release of iron from the ferritin complex [26, 27]. NCOA4 knockout prevents ferritin degradation, leading to ferritin accumulation and reduced release of labile iron. Mitochondrial ferritin sequesters iron within mitochondria, lowering labile iron and oxidative stress, and its upregulation confers protection against ferroptosis [28]. Disruption of iron homeostasis promotes ferroptosis, which may contribute to the progression and destabilisation of atherosclerotic plaques. Excess labile iron drives ROS generation via the Fenton reaction, contributing to oxidative stress and cellular injury. Oxidative stress caused by superoxidation triggers the buildup of oxidised low‐density lipoprotein (ox‐LDL) [29]. Upon interaction with TLR4 on macrophages, Ox‐LDL triggers the NF‐κB pathway, fostering inflammation and increasing intracellular iron levels through upregulation of iron import [30]. Activation of the TLR4/NF‐κB signalling pathway promotes ferroptosis by enhancing oxidative stress and LPO. Conversely, ferroptotic cell death may activate TLR4 in bystander cells through the release of DAMPs, thereby amplifying pro‐inflammatory cytokine expression. Divalent iron overload may trigger the Fenton reaction, driving the overproduction of ROS, exacerbating oxidative stress and LPO, and causing ferroptosis. Upon penetrating impaired endothelial barriers, LDL cholesterol can undergo oxidation. The uptake of this oxidised LDL by macrophages drives foam cell formation [9]. Foam cell buildup within atherosclerotic lesions contributes to the expansion of the lipid core, augmenting LPO, and elevating ROS and malondialdehyde (MDA) levels [31]. Upon activation, macrophages secrete inflammatory cytokines that amplify the vascular inflammation, facilitate lipoprotein oxidation, and accelerate atherosclerosis progression by promoting plaque accumulation [32].

### 3.2 Lipid Metabolism and Ferroptosis

LPO is the oxidative breakdown of lipids, particularly PUFAs, which are key components of membrane phospholipids [13]. In contrast to monounsaturated fatty acids (MUFAs), PUFA‐containing phospholipids are particularly vulnerable to peroxidative damage, owing to the reduced strength of their C—H bonds [33]. Through the exchange of fatty acid chains, the Lands cycle plays a critical role in dynamically remodelling membrane phospholipids, facilitating PUFA regulation through enzymes such as ACSL4 and LPCAT3 [34]. Reducing the expression of the ACSL4 markedly decreases ferroptotic cell death, indicating its role in the ferroptosis pathway linked to lipid oxidation. Through its promotion of ferroptosis, ACSL4 exacerbates tissue damage, which stimulates the release of pro‐inflammatory cytokines and amplifies inflammation [35]. Moreover, ACSL4 facilitates the conversion of long‐chain PUFAs, including AdA or AA, into PUFA‐CoA derivatives through CoA conjugation [20]. After diffusing into cells, these fatty acids are processed by ACSL4 into AA/AdA‐CoA and subsequently esterified into AA/AdA‐PE via LPCAT3. AA/AdA‐PE is subjected to iron‐driven lipid oxidation, occurring through the Fenton reaction or enzymatic pathways. The Fenton reaction, marked by the coupling of Fe^2+^ with hydrogen peroxide, yields highly reactive hydroxyl radicals. These ROS have the potential to inflict damage on cellular lipids, nucleic acids, and proteins. Reacting with PUFAs, hydroxyl radicals create lipid peroxides that pose significant toxicity to cellular membranes. The enzyme ALOX15 specifically oxygenates PUFAs within phospholipids, generating phospholipid hydroperoxides (PLOOH) [36]. The degradation of PLOOHs generates highly reactive and toxic products such as MDA and 4‐hydroxynonenal (4‐HNE), which accumulate and disrupt membrane integrity. 4‐HNE, a principal product of LPO, amplifies oxidative stress and signalling cascades that deteriorate lipid integrity and induce ferroptosis. Superoxide anions produced by mitochondrial dysfunction and NADPH oxidase (NOX) activation are dismutated by SOD into hydrogen peroxide, which is further converted into hydroxyl radicals through the Fenton reaction. Hydroxyl radicals trigger non‐enzymatic LPO by extracting a bisallylic hydrogen atom from PUFA‐PLs within lipid bilayers, thus forming phospholipid radicals. The phospholipid radical undergoes oxidation by oxygen to yield a phospholipid peroxyl radical, which abstracts hydrogen from PUFA, yielding PLOOH. By interacting with PUFA‐PLs, these hydroperoxides, along with radicals such as phospholipid peroxyl radical and alkoxyl radical, extend the chain reaction of PLOOH formation, contributing significantly to the accumulation of 4‐HNE and MDA. Nonheme iron‐containing enzymes such as cyclooxygenase‐2 (COX2), LOXs, and cytochrome P450 reductase can catalyse the oxidation of PUFA‐PLs into PLOOH. ACSL3 activates MUFAs for incorporation into membrane phospholipids, while stearoyl‐CoA desaturase 1 (SCD1) promotes MUFA synthesis. These processes enhance membrane resistance to peroxidation and confer protection against ferroptosis [34]. Furthermore, fatty acyl‐CoA reductase 1 (FAR1) facilitates the conversion of saturated fatty acids (SFAs) into fatty alcohols (FALs), aiding in the biosynthesis of PUFA‐ePLs. Lipid droplets (LDs) are transported to lysosomes for breakdown, liberating free fatty acids, while the buildup of LDs intracellularly is noted to confer antioxidant properties against LPO [37]. Through RAB7A‐mediated lipophagy, the breakdown of LDs can markedly facilitate the onset of ferroptosis [38].

### 3.3 Redox Imbalance and Ferroptosis

Despite the potential harm from oxidative stress, cells have evolved multiple antioxidant defences to lessen LPO and suppress ferroptosis, ensuring a balance between oxidative injury and protective antioxidant function to maintain cellular homeostasis [39]. The primary antioxidant pathway regulating ferroptosis is the System Xc‐/GSH/GPX4 axis, complemented by additional systems including GCH1/BH4, DHODH/CoQ10 and FSP1/CoQ10. In atherosclerosis, the System Xc‐/GSH/GPX4 pathway inhibits lipid peroxide accumulation, thereby protecting VSMCs and VECs from ferroptosis and sustaining plaque stability [4]. Any disturbance in this pathway can lead to heightened LPO, promoting ferroptosis and worsening vascular damage. The FSP1/CoQ10 pathway constitutes a GPX4‐independent ferroptosis defence system in which FSP1 (AIFM2), a FAD‐dependent oxidoreductase, catalyses the NADPH‐dependent reduction of membrane‐embedded coenzyme Q10 (ubiquinone) to ubiquinol (CoQ10H_2_). Ubiquinol acts as a lipophilic antioxidant by scavenging lipid peroxyl radicals, thereby preventing the propagation of LPO [40, 41]. The mitochondrial DHODH/CoQ10 axis protects against ferroptosis independently of GPX4 by catalysing the reduction of CoQ10 to ubiquinol, thereby preventing mitochondrial LPO [10, 42]. The GCH1/BH4 axis is a potent endogenous suppressor of ferroptosis that operates independently of the canonical GPX4/glutathione system. Overexpression of GCH1, the rate‐limiting enzyme in BH4 biosynthesis, increases intracellular BH4 levels. BH4 can scavenge lipid peroxyl radicals, thereby interrupting the propagation of LPO. BH4 promotes CoQ10 biosynthesis and drives MUFA enrichment in phospholipids, thereby conferring resistance to ferroptosis. The GCH1/BH4 axis also suppresses the esterification of PUFAs into phospholipids, thereby depleting peroxidation‐sensitive PUFA‐PLs [10, 43]. These pathways provide complementary defences that protect cells from oxidative stress and suppress the progression of atherosclerosis.

Human atherosclerotic plaques, especially those considered vulnerable to rupture, manifest several molecular markers indicative of ferroptosis [10, 24, 44]. These include markedly reduced GPX4 expression, excessive iron accumulation, and a dysregulated iron homeostasis profile characterised by upregulated transferrin receptor (TFRC/TfR1) and downregulated ferroportin (SLC40A1/FPN) [9, 45, 46]. Vulnerable atherosclerotic plaques exhibit marked accumulation of LPO products, including 4‐HNE and MDA, which are closely associated with plaque instability and the risk of rupture [47, 48, 49]. Although ferroptosis‐related biomarkers show promise in reflecting LPO and iron dysregulation, their clinical translation remains hindered by several critical limitations, including inherent lack of specificity, vulnerability to systemic confounders, poor correlation with intraplaque pathology, insufficient validation in large and diverse patient cohorts, and the confounding influence of disease progression dynamics and plaque heterogeneity.

## 4 Ferroptosis in the Pathogenesis of Atherosclerosis

**FIGURE 2:**
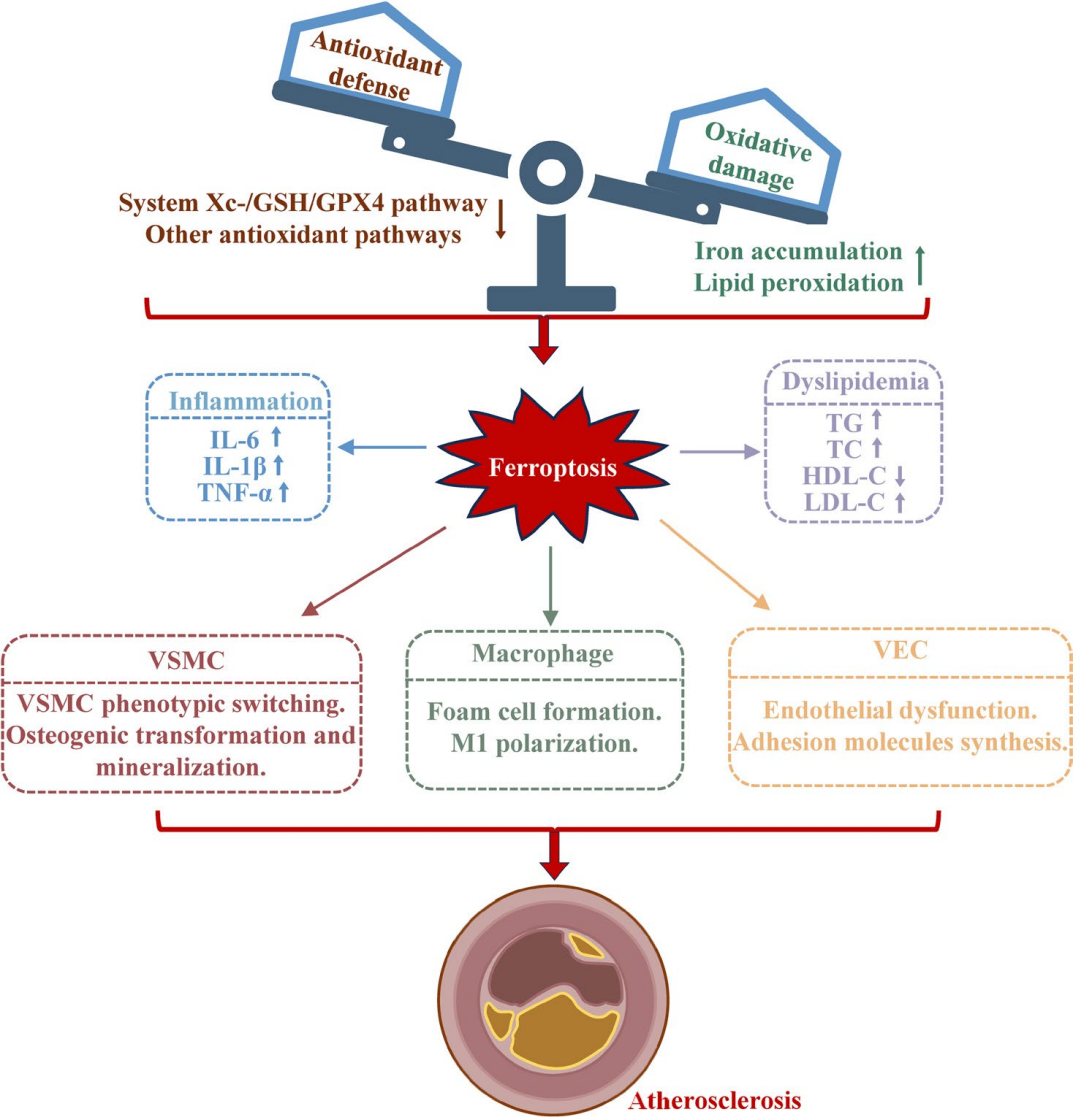
The implications of ferroptosis in atherosclerosis. Ferroptosis, an outcome of the disparity between oxidative harm and antioxidant protection, is significantly involved in various plaque cell activities. These include the induction of a synthetic phenotype in VSMCs, the augmentation of foam cell accumulation, and the exacerbation of endothelial cell dysfunction and inflammation. Additionally, ferroptosis contributes to the release of pro‐inflammatory cytokines and lipid dysregulation. Given the multifaceted roles in the initiation and progression of atherosclerosis, ferroptosis in plaque cells emerges as a central driver of atherogenesis. HDL‐C: high‐density lipoprotein cholesterol; LDL‐C: low‐density lipoprotein cholesterol; TC: total cholesterol; TG: triglycerides; VEC: vascular endothelial cells.

Ferroptosis arises from an imbalance between oxidative injury and antioxidant defences (Figure 2) [50]. This process influences plaque cell dynamics by facilitating VSMC phenotypic switching, foam cell formation, endothelial dysfunction, and enhanced inflammation. Moreover, ferroptosis facilitates the release of pro‐inflammatory mediators and aggravates dyslipidemia. Iron overload induces platelet activation and promotes their aggregation. Additionally, NTBI accelerates LDL oxidation and alters the activity of VECs, VSMCs, and macrophages, thereby promoting atherosclerosis [51].

### 4.1 VEC Dysfunction

VEC impairment represents an early event in the pathogenesis of atherosclerosis [52]. Endothelial dysfunction increases the permeability of the intima and enhances leukocyte adhesion, thereby promoting atherosclerosis progression and thrombus formation. Iron overload triggers endothelial cell damage through its oxidative and inflammatory activities. An overload of free iron ions, beyond what transferrin can bind, can cause endothelial dysfunction. This process facilitates LDL oxidation, impairs nitric oxide synthesis in endothelial cells, elevates ROS, promotes monocyte adhesion, and induces endothelial injury. These processes elevate vascular permeability and induce oxidative injury to the vascular endothelium [53]. Moreover, ox‐LDL can induce endothelial ferroptosis, contributing to atherosclerosis through endothelial dysfunction and inflammation. Blocking ferroptosis mitigated endothelial injury and LPO triggered by ox‐LDL. The accumulation of NTBI due to iron overload not only accelerates LDL oxidation but also induces endothelial dysfunction [54].

### 4.2 Dysfunction of VSMC

Dysfunction of VSMCs and their participation in ferroptosis are gaining recognition as key elements in the pathogenesis of atherosclerosis. As the predominant cellular component of the arterial media, VSMCs are critical for proper vascular function and stability [55]. The migration of VSMCs to the intima contributes to the initial development of atherosclerotic plaques. In VSMCs, reduced GPX4 activity or compromised antioxidative defences can render cells more prone to ferroptosis, particularly within the inflammatory setting of atherosclerotic plaques. The release of signalling molecules from dying VSMCs contributes to atherosclerosis by amplifying inflammatory pathways [56]. Excessive iron impairs VSMC function, leading to dysregulation of apoptosis, aberrant proliferation, phenotypic transformation, enhanced ROS generation, and vascular calcification. Iron‐driven free radicals lead to the intracellular buildup of oxidised LDL in smooth muscle cells. Oxidative stress triggered by ox‐LDL, along with ROS production and iron buildup, can synergistically drive ferroptosis in VSMCs. Excessive iron and impaired antioxidant mechanisms promote ferroptosis, thereby inducing extensive VSMC injury. During atherosclerosis progression, VSMCs proliferate and migrate to the intima, thereby contributing to fibrous cap formation and plaque stabilisation [57]. Phenotypic switching of VSMCs can promote inflammatory responses and enhance extracellular matrix synthesis, thereby contributing to plaque stabilisation [57, 58]. However, in advanced atherosclerotic lesions, excessive VSMC death compromises the integrity of fibrous caps, heightening the risk of plaque rupture.

### 4.3 Macrophage Dysfunction

Iron overload exerts a profound influence on macrophage function and is a key driver of atherosclerotic pathogenesis [30]. Iron accumulation modifies the metabolic profile of macrophages, enhancing glycolysis to satisfy the energetic and synthetic demands associated with inflammatory and proliferative events within atherosclerotic plaques [59]. Dysfunctional macrophages exhibit reduced efficiency in clearing oxidised LDL, facilitating foam cell formation and its accumulation within the arterial walls. Hepcidin, a pivotal modulator of iron homeostasis, controls iron efflux from macrophages and contributes to atherosclerosis progression. The characteristics and abundance of macrophages within atherosclerotic plaques are important factors in the advancement of atherosclerosis. Moreover, macrophages activated by lipopolysaccharides through TLR4 exhibit increased lipid uptake and impaired cholesterol uptake, thereby promoting lipid accumulation and foam cell formation within atherosclerotic plaques [60]. Furthermore, macrophages secrete lipid mediators, including leukotrienes and prostaglandins, which serve to intensify the inflammatory response. ROS produced by macrophages contribute to oxidative stress, instigating arterial wall damage and fuelling chronic inflammation. Iron overload in macrophages weakens their antioxidant capacity and alters the secretion of cytokines. Macrophages release inflammatory mediators such as IL‐1β, IL‐6, and TNF‐α, which amplify inflammatory responses, recruit more immune cells, and sustain continued cytokine production. By secreting MCP‐1, macrophages orchestrate the recruitment of monocytes to atherosclerotic plaques. The differentiation of these monocytes into macrophages sustains the inflammatory response and promotes lipid uptake [61]. Iron overload stimulates macrophage inflammation by enhancing the association of 5‐LOX with the nuclear membrane. In later stages of atherosclerosis, macrophages release various inflammatory mediators and matrix metalloproteinases, causing collagen degradation in the fibrous cap and promoting VSMC apoptosis, further destabilising the plaque [62].

## 5 Interaction Between Macrophages and Ferroptosis

The overlapping features of ferroptosis and macrophage functions strongly point to a potential relationship [63]. During ferroptosis, the accumulation of iron and ROS contributes to LPO and the release of various inflammatory mediators [64]. Oxidised phospholipids and damage‐associated molecular patterns (DAMPs) released from ferroptotic cells can be recognised by macrophages through pattern recognition receptors such as Toll‐like receptor 2 (TLR2). Recognition of oxidised PUFA‐PLs by TLR2 facilitates macrophage‐mediated engulfment and clearance of ferroptotic cells [65]. Upon encountering ferroptotic debris, macrophages are activated and secrete pro‐inflammatory cytokines, including IL‐6, TNF‐α and CCL2, amplifying the local inflammatory response [66]. During this process, high‐mobility group box 1 (HMGB1) released from ferroptotic cells binds to the advanced glycosylation end‐product receptor (AGER/RAGE) on macrophages, further stimulating cytokine production [67]. Chemokines such as CCL2 and CCL7 recruit macrophages to the ferroptotic sites, thereby amplifying the local inflammatory response. Additionally, iron liberated from ferroptotic cells or heme catabolism augments macrophage intracellular iron stores. Excess iron promotes ROS and LPO, intensifying macrophage activation and inflammatory signalling. IL‐6 and TNF‐α may further sensitise cells to ferroptosis by disturbing iron balance and promoting oxidative stress. Collectively, ferroptosis establishes a pro‐inflammatory microenvironment that activates macrophages and reinforces local inflammation.

Macrophages, as key immune regulators, display distinct polarisation states that influence ferroptosis [68]. The classical M1 phenotype, induced by pathogens or IFN‐γ, is characterised by high ROS production, nitric oxide synthase (iNOS) activity, and secretion of pro‐inflammatory cytokines such as IL‐1β, IL‐6 and TNF‐α. In contrast, M2 macrophages, stimulated by Th2 cytokines such as IL‐4 and IL‐13, secrete anti‐inflammatory mediators such as IL‐10, TGF‐β, PDGF and FGF that promote tissue repair [68, 69]. Iron metabolism plays a pivotal role in this polarisation process. M1 macrophages exhibit elevated ferritin expression and reduced ferroportin levels, favouring intracellular iron accumulation, while M2 macrophages display high FPN and CD163 expression, supporting iron export [70]. Iron overload enhances glycolytic metabolism and promotes M1 polarisation, thus amplifying inflammatory responses and susceptibility to ferroptotic stress [71]. Conversely, the antioxidant environment of M2 macrophages confers relative resistance to ferroptosis. Interestingly, M1‐polarised macrophages can exhibit partial resistance to ferroptosis, likely due to metabolic rewiring and compensatory antioxidant mechanisms [72]. Under physiological conditions, macrophages contribute to tissue homeostasis through the clearance of ferroptotic cells. Nevertheless, excessive ferroptotic stress can lead to macrophage dysfunction and impaired immune responses. Notably, activated macrophages, particularly the M1 subtype, produce a pro‐inflammatory microenvironment characterised by high iron content, elevated ROS, and cytokine release, which can in turn enhance ferroptotic susceptibility in adjacent cells.

Emerging evidence reveals that disturbed macrophage iron trafficking, inflammatory polarisation, and defective efferocytosis are not independent contributors to plaque progression but form a self‐reinforcing pathogenic circuit that promotes LPO, ferroptotic cell death, and necrotic core expansion [10, 30]. Dysregulation of the hepcidin‐FPN axis increases intracellular iron retention in plaque macrophages by reducing FPN‐mediated iron export [73]. Dysregulated NCOA4‐dependent ferritinophagy mobilises ferritin iron into the labile iron pool. The resultant elevation of the labile iron pool drives Fenton‐mediated ROS production and LPO, creating a pro‐ferroptotic milieu [28, 74]. Excess labile Fe^2+^ in lipid‐laden foam cells drives the iron‐dependent decomposition of polyunsaturated fatty acid‐containing phospholipid hydroperoxides (PUFA‐PL‐OOH) into reactive lipid radicals, thereby propagating LPO chain reactions. In the context of impaired GPX4 function, accumulation of these toxic lipid peroxides executes ferroptotic cell death. M1 macrophages actively accumulate intracellular iron to sustain their proinflammatory functions by upregulating iron‐import machinery while downregulating the iron exporter ferroportin. The resulting elevation in the labile iron pool serves not only as a cofactor for metabolic demands but also as a signalling molecule that further reinforces M1 polarisation through activation of proinflammatory pathways [75]. Excess free iron also catalyses LPO, causing damage to cellular membrane systems and ultimately triggering ferroptotic cell death. Moreover, M1 macrophages inherently exist in a highly oxidative environment and produce substantial amounts of ROS [76]. Combined with their propensity for iron accumulation, this positions M1 macrophages as potentially more susceptible to ferroptosis than M2 macrophages in iron‐overloaded inflammatory milieus, such as atherosclerotic plaques. Both the proinflammatory M1 phenotype and ferroptosis markedly impair efferocytosis, leading to defective clearance of dying cells and consequent secondary necrosis [63]. This process releases additional redox‐active iron into the plaque microenvironment, creating a self‐reinforcing loop that amplifies oxidative and inflammatory stress, drives necrotic core expansion, and increases plaque vulnerability in atherosclerosis [12, 77].

## 6 Macrophage Dynamics in Atherosclerosis

**FIGURE 3:**
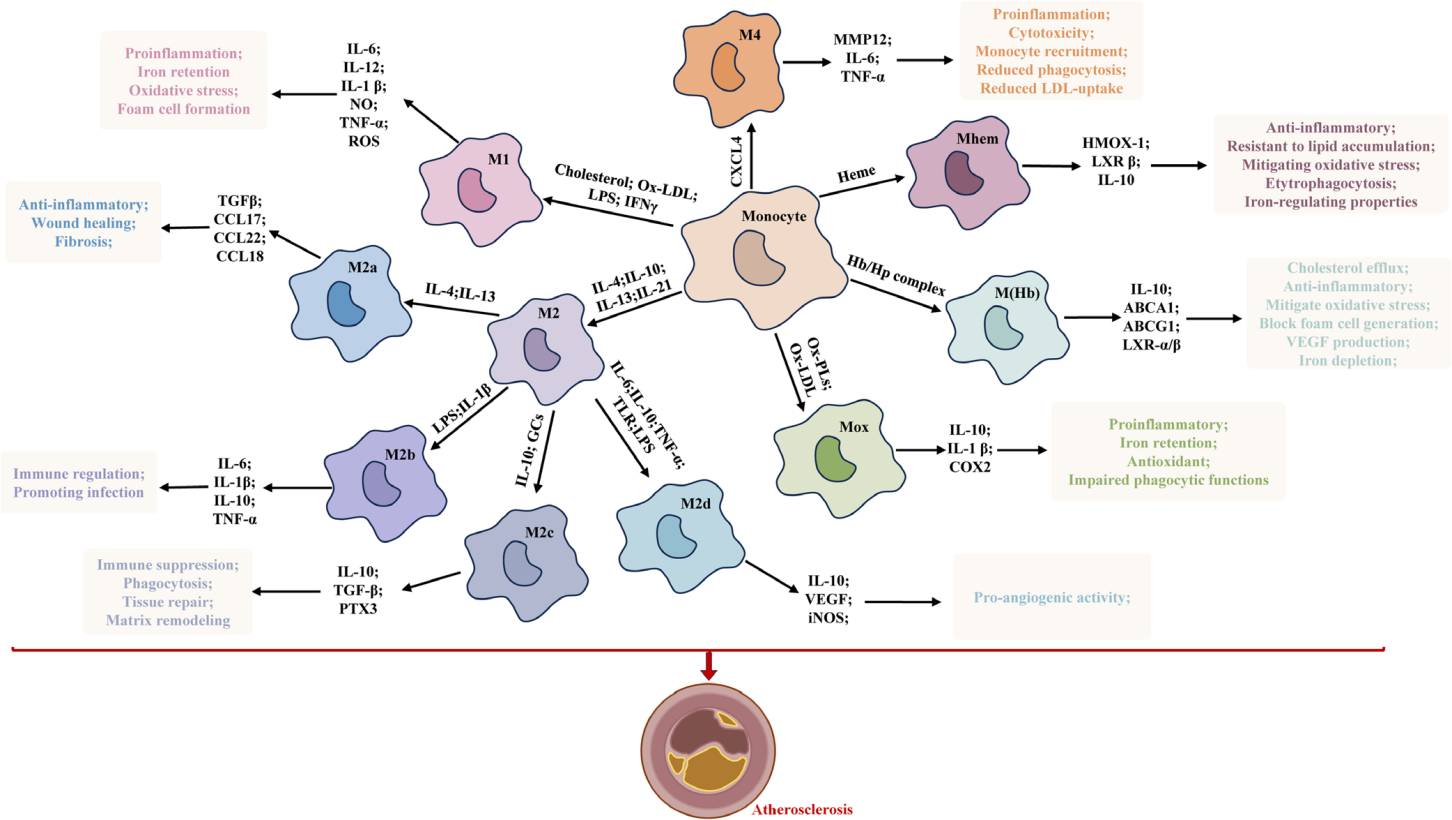
Macrophage phenotypes within atherosclerotic plaques. The milieu of atherosclerotic plaques influences the transformation of monocytes into diverse macrophage types. GCs: glucocorticoids; Hb: haemoglobin; Hp: haptoglobin; LPS: lipopolysaccharide; OxPLs: oxidised phospholipids.

The evolution of atherosclerosis critically hinges on macrophage function, with these cells orchestrating events from the initial formation of plaques to the development of necrotic cores, and finally, participating in the resolution of the lesion [75, 78]. Dysregulated iron and lipid homeostasis are considered significant determinants in the pathogenesis and advancement of atherosclerosis. IFIT2 expression was markedly elevated in atherosclerotic lesions and foam cells. Moreover, silencing IFIT2 attenuated the ox‐LDL‐driven elevation of ferritin proteins and iron content within foamy macrophages. Furthermore, IFIT2 silencing also mitigated iron retention and lipid build‐up in atherosclerosis lesions, promoting cholesterol efflux from foam cells via activation of the PPARγ/LXRα/ABCA1‐ABCG1 axis [79]. Macrophage‐derived iron accumulation within plaques critically influences the progression of atherosclerosis. The interaction of iron and lipid metabolic pathways predominantly occurs within macrophage‐rich atherosclerotic lesions, where macrophages accumulate excess lipid and iron, thereby promoting plaque progression. Fpn1 deficiency leads to iron buildup within plaque macrophages, inducing local ROS production and accelerating lesion progression. In response to haemoglobin stimulation, macrophages exhibit diminished intracellular iron levels through increased ferroportin expression, subsequently attenuating oxidative stress caused by iron and potentiating ABCA1 and ABCG1 expression via LXRα. Conversely, iron accumulation caused by Fpn1 deficiency hampers ABC transporter activity by suppressing LXRα levels [80]. Atherosclerotic lesions contain a complex cellular landscape that directs monocyte differentiation into functionally distinct macrophage phenotypes. Within these lesions, macrophages form a heterogeneous population comprising both pro‐inflammatory and anti‐inflammatory subtypes. An imbalance favouring pro‐inflammatory macrophages contributes to the pathogenesis of atherosclerosis through the exacerbation of inflammation and promotion of plaque growth. M1 macrophages exhibit a robust capacity for ROS generation, primarily through enhanced NOX activity [32]. When exposed to ROS, LDL undergoes oxidative alteration to yield ox‐LDL. Macrophage surface receptors promptly internalise ox‐LDL, driving foam cell formation, which signifies the onset of atherosclerosis. M1 macrophage activity has been associated with plaque instability and an increased risk of rupture [81]. The M2 and Mhem macrophage phenotypes show a reduced propensity for lipid accumulation, possess iron‐regulating capacity, and exert anti‐inflammatory activities [76, 82]. M2 macrophages primarily participate in anti‐inflammatory and tissue‐reparative processes. Their accumulation is associated with organised macrocalcification and fibrous cap formation, features that characterise stable atherosclerotic plaques. Plaque stabilisation correlates with a greater abundance of M2 macrophages in comparison to M1 macrophages (Figure 3). M1 macrophages are pro‐atherogenic, characterising progressive lesions and contributing to the development of unstable plaques. During atherosclerosis regression, M2 macrophages promote tissue remodelling and stabilise the atherosclerotic plaque architecture. M2 macrophages mediate cholesterol export from foam cells and enhance the removal of apoptotic cells via increased efferocytosis within atherosclerotic sites. M2a macrophages can be polarised by Th2 cytokines, such as IL‐13 and IL‐4, with their primary functions centred around promoting tissue regeneration and dampening inflammation. As regulatory cells, M2b macrophages are activated upon exposure to immune complexes, initiating TLRs or IL‐1 receptor signalling that mediates the immunomodulatory activity [83]. M2c macrophage polarisation can be induced by TGF‐β, glucocorticoids, and IL‐10, resulting in the upregulated expression of anti‐inflammatory cytokines and chemokines, such as CCL16, IL‐10, CCL18, and TGF‐β, which enhances the phagocytosis of apoptotic cells. M2d macrophages reduce inflammation within plaques through the production of anti‐inflammatory cytokines. M2d macrophages orchestrate fibrous cap formation through the synthesis of collagen and extracellular matrix constituents. Additionally, they exhibit robust phagocytic activity, efficiently clearing apoptotic cells and debris within the plaque, thereby promoting the resolution of inflammation and stabilising the atherosclerotic lesion. M2d macrophages exhibit elevated IL‐10 and VEGF production but diminished IL‐12 and TNF levels, thereby promoting angiogenesis and mitigating lipid buildup. Mhem macrophages exert protective influences against atherosclerosis through CD163‐mediated phagocytosis of erythrocytes and haemoglobin‐haptoglobin (Hb:Hp) complexes, leading to upregulation of ferroportin and heme oxygenase‐1 (HO‐1). These changes promote iron export and antioxidant defence, thereby inhibiting foam cell formation. Mox macrophages exhibit an antioxidant gene signature but display impaired phagocytic activity and limited reparative function, which may contribute to plaque progression. M4 macrophages exhibit impaired phagocytic activity and produce elevated levels of pro‐inflammatory cytokines, potentially contributing to plaque progression [84]. LDL infiltrates the vascular intima via compromised endothelial cells and undergoes oxidation to form ox‐LDL, which subsequently stimulates the release of chemokines and adhesion molecules that attract circulating monocytes. Within a local setting characterised by pro‐inflammatory cytokines and growth factors, monocytes undergo differentiation into macrophages. During the onset of atherosclerosis, M1 macrophages consume oxidised LDL, transforming into foam cells. Apoptotic M1‐derived foam cells that escape timely efferocytosis may undergo secondary necrosis, leading to the accumulation of the necrotic core and increasing the potential for plaque rupture [62]. During regression, M2 macrophages facilitate cholesterol efflux and promote the clearance of apoptotic cells through efferocytosis, contributing to plaque stabilisation [61].

## 7 Macrophage Ferroptosis in Atherosclerosis

**FIGURE 4:**
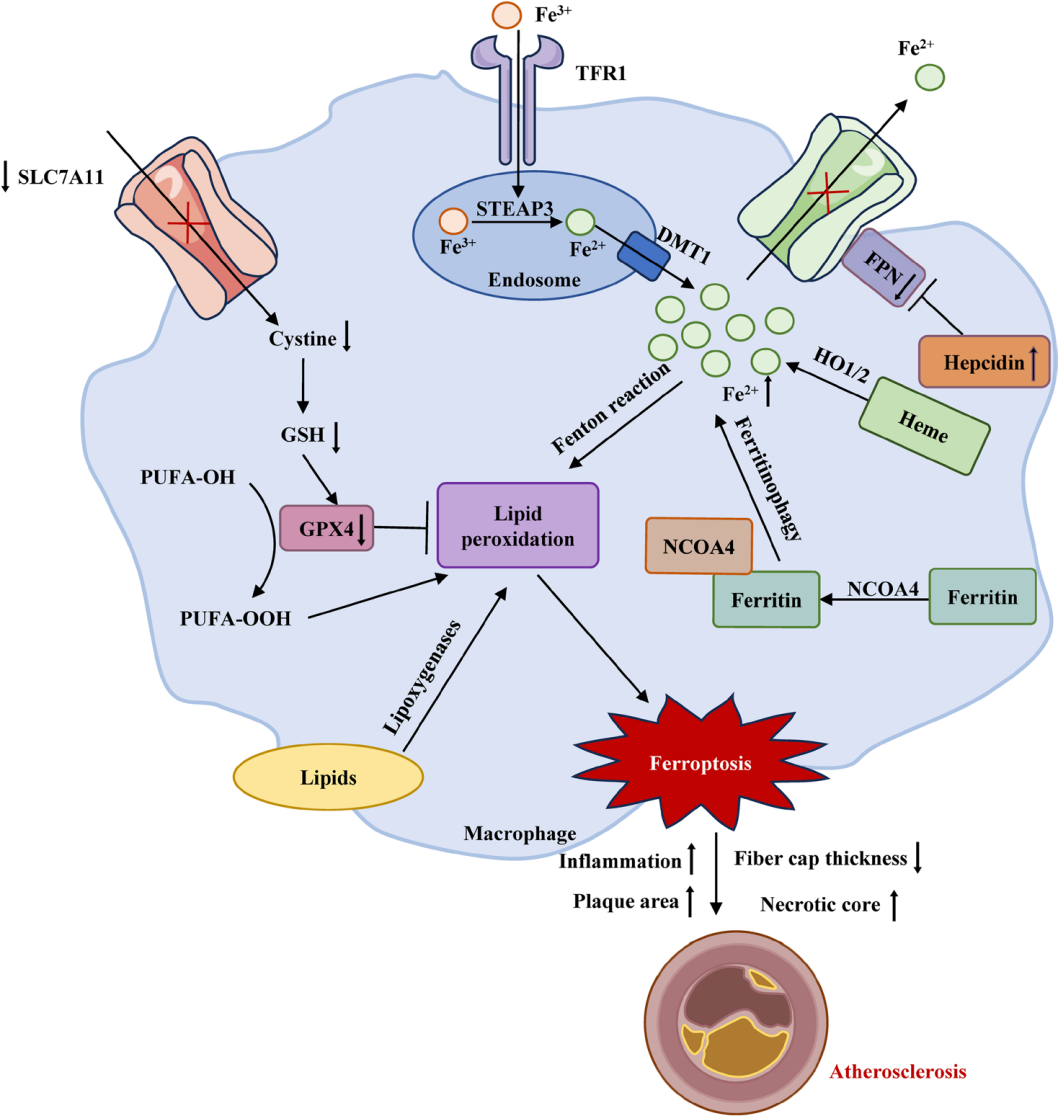
The influence of macrophage ferroptosis on atherosclerosis. Overaccumulation of ROS or iron within macrophages promotes lipid peroxidation and other detrimental processes, culminating in macrophage ferroptosis and thereby amplifying plaque instability. FPN: ferroportin; ROS: reactive oxygen species.

Atherosclerosis represents a chronic ailment typified by the buildup of lipids and the generation of fibrous lesions in the intima of arteries, causing vessel narrowing and increased rigidity (Figure 4) [85, 86]. Erythrocytes are destroyed and accumulate within atherosclerotic plaques. Their subsequent phagocytosis by macrophages results in iron overload within these cells. Perturbations in macrophage homeostasis, particularly an overabundance of ROS or iron overload, promote LPO, thus promoting ferroptosis and exacerbating plaque instability. Macrophage ferroptosis can influence plaque stability and may contribute to necrotic core enlargement. The NOD1/CXCR2 pathway crucially modulates macrophage motility and is implicated in the pathogenesis of atherogenesis. Spleen‐dependent NOD1 activation exerts a significant influence on iron metabolism and macrophage recruitment. Stimulation of NOD1 may modulate ferroptosis‐related pathways in macrophages, potentially influencing GPX4 expression [87]. Elevated ALOX5 expression is linked to both LPO and inflammatory responses, potentially serving as a driver of ferroptotic processes in macrophages. NCF2, a constituent of the NOX machinery, participates in the formation of ROS, which drives oxidative stress and LPO, thereby potentiating macrophage ferroptosis and impairing plaque stability. NCF2 and ALOX5 potentially drive necrotic core development in atherosclerosis through modulating macrophage ferroptosis [88]. By engulfing ox‐LDL through phagocytosis, macrophages amass significant amounts of lipids in their cytoplasm, thus transforming into foam cells. Moreover, ox‐LDL in atherosclerotic plaques is taken up by macrophages through TLR4, activating NF‐κB. This elevates hepcidin levels, promotes ferroportin degradation, and exacerbates iron accumulation. Meanwhile, the elevation of ATF3 decreases SLC7A11, thus diminishing the GPX4/GSH antioxidant pathway and encouraging ferroptosis within macrophages [24]. Ox‐LDL markedly amplifies ferroptosis along with IDH1 protein expression within macrophages. Ferrostatin‐1 can diminish the rise in Fe^2+^ caused by ox‐LDL, decrease LPO and lactate dehydrogenase, and prevent GSH reduction. Concurrently, it promotes the upregulation of GPX4, FTH1, and SLC7A11, reinforcing cellular defence mechanisms. Blocking IDH1 mitigates Fe^2+^ overload, GSH depletion, lactate dehydrogenase levels, and LPO, while elevating GPX4, FTH1, and SLC7A11 levels, thus decreasing the ferroptosis driven by ox‐LDL in macrophages [89]. Elevated uric acid has a specific regulatory effect on ox‐LDL‐elicited ferroptosis in macrophages, and accelerates foam cell development via ferroptotic mechanisms. Moreover, it provokes mitochondrial impairment, which is instrumental in inducing ferroptosis in macrophage‐derived foam cells. In addition, it accelerates atherosclerosis by altering NRF2‐dependent autophagy and causing ferroptosis. In macrophages within atherosclerotic lesions, it also reduces the expression of GPX4, SLC7A11, and NRF2 [90]. The accumulation of lipid peroxides, iron, and macrophages within coronary lesions implicates an augmented incidence of macrophage ferroptosis in atherosclerosis. ApoA1 amplifies System Xc‐, resulting in elevated intracellular GSH and diminished ferroptosis. ApoA1 stimulates NRF2, a critical regulator of SLC7A11 expression, by facilitating its migration into the nucleus. Hence, HDL/ApoA1 curtails macrophage ferroptosis via enhancing the NRF2/SLC7A11/GSH pathway [91]. JAK2^V617F^ mice, marked by increased levels of platelets, white blood cells, and RBCs, manifest hastened atherosclerosis and macrophage‐mediated erythrophagocytosis. VFEpoR RBCs exhibit compromised antioxidant defences and elevated lipid hydroperoxide levels, and their phagocytosis drives the induction of macrophage ferroptosis. Specifically, in M2 macrophages, phagocytosis of VFEpoR RBCs diminished GPX4 levels and augmented the formation of MDA, a byproduct of LPO [92]. Macrophages undergoing ferroptosis secrete inflammatory chemicals like IL‐1β, TNF‐α, and IL‐6, drive the synthesis of MMP‐9 and MMP‐2, and discharge cellular contents and lipids, promoting necrotic core formation and accelerating atherosclerosis [93]. IL‐23p19 contributes to the genesis and advancement of atherosclerosis, and its knockout could decrease M1 macrophage polarisation, thus enhancing cardiac remodelling by mitigating ferroptosis in macrophages [94]. GCH1 knockdown elevated levels of iNOS, IL6, TNF‐α, and IL‐1β, and concurrently reduced levels of CD206, IL10. GCH1 can serve as protective modulator against ferroptosis, diminish M1 macrophage differentiation, and moderate inflammation in macrophages exposed to lipopolysaccharide, whereas GCH1 suppression potentiated lipopolysaccharide‐elicited ferroptosis in macrophages and dampened AMPK [95]. Cigarette tar substantially fosters the accumulation of lipid‐laden plaques, exhibiting expanded necrotic cores and diminished fibrous components, driving iron accumulation and LPO within atherosclerotic lesions. Furthermore, cigarette tar notably elevates hepcidin levels while reducing SLC7A11 and ferroportin expression in macrophages within these plaques. Cigarette tar propels atherosclerosis advancement by initiating macrophage ferroptosis through the NF‐κB‐dependent hepcidin/FPN/SLC7A11 axis [96].

## 8 Therapeutic Targeting Macrophage Ferroptosis in Atherosclerosis

Ferroptosis inhibitors, including iron chelators and antioxidants, have been explored as therapeutic strategies for atherosclerosis by stabilising plaques through suppression of ferroptotic cell death (Table 1). Conversely, ferroptosis inducers could theoretically mitigate inflammation by selectively eliminating deleterious macrophages rather than directly stabilising plaques. However, this approach requires precise targeting to prevent collateral damage to healthy cells, and its therapeutic efficacy and safety remain to be rigorously evaluated.

**TABLE 1:** Therapeutic targeting of macrophage ferroptosis in atherosclerosis.

Agent	Agent class	Primary molecular target	Effects in macrophages	Effects in VEC/VSMC/systemic	Therapeutic potential in atherosclerosis
Liproxstatin‐1; ferrostatin‐1	Lipophilic radical‐trapping antioxidants	Lipid ROS/lipid peroxidation	Inhibit LPO; suppress macrophage ferroptosis; reduce macrophage‐driven inflammation and necrotic core expansion	Protect VECs/SMCs from ferroptosis; preserve endothelial barrier; reduce vascular oxidative stress	Macrophage stabilisation; attenuated intraplaque inflammation; promoted plaque stability
Deferoxamine; deferasirox	Iron chelators	Labile iron pool	Reduce labile iron; inhibit Fenton‐driven ROS; decrease macrophage ferroptosis and foam cell formation; reduce iron‐driven M1 polarisation	Reduce iron‐induced EC/SMC dysfunction; systemic hypoferremia and hepcidin alterations	Reduction of plaque iron burden and oxidative stress; caution for systemic iron deprivation
Baicalein; Loxblock‐1/2/3	LOX inhibitor	12/15‐LOX	Decrease LOX‐mediated LPO; suppress macrophage inflammatory activation and ferroptosis	Inhibit LOX activity in ECs/VSMCs; reduce endothelial dysfunction; broad antioxidant effects possible	Attenuation of macrophage LPO and inflammation; slowed plaque progression

Abbreviations: LPO: lipid peroxidation; VEC: vascular endothelial cell; VSMC: vascular smooth muscle cell.

Iron chelators, such as deferasirox and deferoxamine, sequester labile iron, thereby reducing its availability for Fenton reactions that yield ROS. Lowering macrophage iron via iron chelators can reduce LPO and ferroptosis [6, 17]. Blocking macrophage ferroptosis via iron chelators may stabilise atherosclerotic plaques, reduce rupture risk and slow disease progression [97]. Integrating iron chelation with anti‐inflammatory or antioxidant macrophage therapies may offer a therapeutic avenue for atherosclerosis management. Lipophilic antioxidants like liproxstatin‐1 and ferrostatin‐1 have emerged as promising therapeutic agents in atherosclerosis due to their ability to counter the deleterious consequences of macrophage ferroptosis [98]. By inhibiting ROS accumulation and preventing LPO, these agents protect macrophages from ferroptosis, preserve macrophage survival, suppress inflammatory cytokine production, lessen the expression of oxidative stress markers, and promote plaque stabilisation [99]. Pharmacological inhibition of ferroptosis using liproxstatin‐1 led to a significant reduction in lesional 4‐HNE, iron accumulation, macrophage TfR expression, and erythrophagocytosis. Liproxstatin‐1 diminished endothelial hyperpermeability in VFEpoR mice, indicating its potential to reverse ferroptosis‐driven plaque progression [92]. LOX inhibitors such as baicalein and the experimental agents Loxblock‐1/2/3 suppress LOX activity, thereby decreasing LPO and the production of inflammatory signals, which could halt macrophage ferroptosis and potentially slow the advancement of atherosclerotic plaques [77]. Micheliolide, a derivative of parthenolide, exhibits antioxidant and anti‐inflammatory properties. It ameliorates atherosclerosis in ApoE^−/−^ mice by enhancing lipid profiles and dampening inflammation. Micheliolide elevates xCT and GPX4 through NRF2 activation, boosts mitochondrial activity, alleviates oxidative stress, and diminishes LPO, thereby blocking macrophage ferroptosis triggered by ox‐LDL. Micheliolide mitigates atherosclerosis through its influence on the KEAP1/NRF2 pathway, diminishing macrophage ferroptosis [5]. Tricetin exhibits notable anti‐inflammatory and antioxidant properties. It elevates xCT and GPX4, mitigates oxidative stress, enhances mitochondrial efficiency, and blocks LPO, thus countering ferroptosis in macrophages caused by ox‐LDL. Tricetin facilitates NRF2 nuclear translocation, leading to ferroptosis suppression via the NRF2 pathway. The NRF2 inhibitor ML385 diminished Tricetin's regulatory effects on oxidative stress modulation and ferroptosis inhibition [100]. Melatonin triggers the NRF2/SLC7A11/GPX4 axis, augmenting antioxidant defences, diminishing lipid oxidation, and downregulating Lp‐PLA2 within macrophages. In macrophages exposed to NRF2 inhibitor ML385 and ApoE^−/−^ mice where NRF2 was blocked through AAV‐sh‐NRF2, the ameliorative effects of melatonin on lipid oxidation, Lp‐PLA2 expression, antioxidant activity, and ferroptosis were notably impaired [101]. The NF‐κB antagonist can protect macrophages from ferroptotic cell death by counteracting tar‐induced disruption of iron homeostasis controlled by the hepcidin/FPN/SLC7A11 axis [96]. Suppression of IDH1 diminishes ox‐LDL‐stimulated ferroptosis and the generation of macrophage foam cells via NRF2 activation, potentially mitigating the progression of atherosclerosis [89]. The BMP inhibitor LDN 193189 downregulated hepcidin gene expression in the liver while enhancing ferroportin levels in macrophages, leading to reduced intracellular iron and hydrogen peroxide levels. This reduction in macrophage iron via LDN 193189 treatment correlated with enhanced ABCG1 and ABCA1 expression, facilitated cholesterol export to ApoA‐1, diminished foam cell production, and mitigated atherosclerosis [73].

Compounds such as erastin inhibit the system Xc‐ antiporter, inducing ferroptosis through GSH depletion and heightened LPO. Moreover, L‐buthionine sulfoximine suppresses γ‐glutamylcysteine synthetase to deplete GSH, increase LPO and promote ferroptotic cell death. By targeting GPX4, RSL3 and altretamine induce ferroptosis, potentially diminishing the viability of pro‐inflammatory macrophages in atherosclerotic environments and contributing to reduced inflammatory burden and slower plaque evolution.

## 9 Conclusion

Atherosclerosis, a chronic inflammatory disorder of the arterial wall, is a major cause of cardiovascular mortality. Macrophages, fundamental to the evolution of atherosclerotic plaques, have emerged as particularly susceptible to ferroptosis. Recent advancements have elucidated the interaction between macrophage ferroptosis, iron metabolism, and atherosclerosis pathogenesis. This review compiles current insights into the intricate link between macrophage ferroptosis and atherosclerosis, highlighting potential therapeutic implications. Dysregulated iron metabolism heightens oxidative stress, thereby driving LPO and redox imbalances that characterise ferroptosis. In atherosclerosis, disrupted iron homeostasis can worsen LPO and amplify inflammatory responses, forming a cycle that destabilises plaques. Ferroptosis is intricately linked with the dysfunction of key vascular cells, including VECs, VSMCs, and macrophages. Macrophage ferroptosis contributes to plaque instability by expanding necrotic cores and intensifying inflammation. Ferroptosis functions not only as a stress response but also as a modulator of macrophage behaviour within the plaque microenvironment. Ferroptotic macrophages can enhance plaque vulnerability through cell death and subsequent inflammatory responses. Therapeutic strategies such as iron chelation and antioxidant augmentation may prevent macrophage death and promote plaque stabilisation. Exploring macrophage ferroptosis offers profound insights into the intricacies of atherosclerosis, unlocking opportunities for new interventions.

## Author Contributions

**Xiehui Chen:** conceptualization, methodology, writing – original draft preparation, writing – reviewing and editing. **Xiangbo Liu:** data curation, investigation, supervision, visualisation, software, writing – original draft preparation. **Changchun Zeng:** conceptualization, funding acquisition, validation, writing – reviewing and editing. All authors approved the final manuscript.

## Funding

This work was supported by the National Natural Science Foundation of China, 82270940.

## Conflicts of Interest

The authors declare no conflicts of interest.

## Data Availability

Data sharing is not applicable to this article as no datasets were generated or analysed during the current study.
